# An exploratory study of creativity and eating disorders

**DOI:** 10.1186/s40337-017-0176-9

**Published:** 2017-10-19

**Authors:** Bruce D. Burns, Yichelle Zhang, Mareike Wieth, Stephen Touyz

**Affiliations:** 1 University of Sydney, Sydney, New South Wales Australia; 2 Albion College, Albion, Michigan USA

## Abstract

**Background:**

We examined whether cognitive rigidity associated with having an eating disorder generalized to creativity.

**Method:**

One hundred twelve participants from the participant pool of an Australian university were given a measure of disordered eating (EDE-Q), asked if they had ever had a diagnosis of an eating disorder (16 reported yes), and given 3 min to generate alternative uses for a paper-clip. The alternative uses task yielded measures of creative fluency, originality, elaboration and flexibility.

**Results:**

A logistic regression found that only lower flexibility predicted a self-reported ED diagnosis. Across the spectrum of disordered eating behaviour there was no association between creativity measures and EDE-Q global scores.

**Conclusion:**

Our results were consistent with previous findings of an association between cognitive inflexibility and having an ED. However we found no evidence that cognitive inflexibility generalized to creativity more broadly. Our results may lend support to Cognitive Remediation Therapy, but further study is required.

## Plain English summary

Previous research has found that people with eating disorders, particularly anorexia, have greater “cognitive rigidity”, as measured by their poor ability to switch between tasks. The term “cognitive rigidity” seems to suggest a general inflexibility of thought, such as limited creativity. We addressed the question “Are people with eating disorders less creative?” by asking them to come up with new uses for a common object. This gives people a chance to display both originality (that is, generating uses few other people come up with) and flexibility (that is, coming up with uses not related to each other). Participants in our experiment were women attending a large Australian metropolitan university, of whom 16 out of 112 reported a current or previous diagnosis of an eating disorder. We found that the diagnosed women showed much lower flexibility than women with a diagnosed eating disorder, but that they did not differ on other measures of creativity such as originality. This is evidence that women with an eating disorder may be less flexible in how they approach a task, consistent with their poor performance when they have to switch tasks, but may not have a general rigidity in how they think.

## Background

### An exploratory of creativity and eating disorders

Eating disorders (ED) such as anorexia nervosa (AN) and bulimia nervosa (BN), are serious mental illnesses characterised by a multitude of factors including restricting dietary intake, excessive exercise, preoccupation with body weight and shape, strong need for perfectionism, and changes in cognitive functioning. Changes in cognitive functioning can be especially problematic as they may be maintenance factors in the continuation of maladaptive eating psychopathology. Schmidt and Treasure [1] propose that cognitive rigidity may be such a factor for EDs. Using a series of set shifting tasks (e.g., the Wisconsin Card Sorting Task) and coherence tasks (e.g., the Rey-Osterriech Complete Figure Task), Harrison, Tchanturia, Naumann and Treasure [2] found that those with AN and BN had poorer performance compared to healthy controls. Poorer set shifting performance has also been found to be associated with illness duration and extent of ED rituals but not with body mass index across the spectrum of ED diagnoses (Roberts, Tchanturia, & Treasure [3]). Furthermore Anderluh, Tchanturia, Rabe-Hesketh and Treasure [4] found that retrospectively reported childhood traits of inflexibility and rigidity were predictive of developing an ED. However a meta-analysis of 22 studies (Westwood, Stahl, Mandy, Tchanturia [5]) found a significant difference in perseveration errors in adults but not in children across a range of AN populations, suggesting that cognitive rigidity develops as a consequence of developing an ED as opposed to being a precursor trait prior to the development of an ED.

Whilst there has been a growing body of literature examining cognitive rigidity in ED populations, the scope of cognitive rigidity has not been clearly articulated. The term “cognitive rigidity” would seem to imply not just difficulty in set shifting, but additionally a more general inflexibility and difficulty in divergent thinking. This would appear to predict that people with an ED would be less creative than otherwise similar individuals. Creativity is sometimes seen as only a characteristic of people who make major artistic or scientific contributions, but Guilford [6] pointed out that people can be regularly creative in small ways. Simonton [7] used the term ‘big-C’ creativity to refer to creative contributions that have a major impact on society. On the other hand, ‘little-C’ creativity is the kind of creativity one might see on a daily basis when someone adapts to change or comes up with new ways of understanding a problem. Guilford argued that at the heart of creativity is a process called *divergent production*, now more often referred to as *divergent thinking.* Guilford contrasted this with intelligence tests that focus on convergence on a single correct answer, whereas divergent thinking is the ability to generate new information or solutions from given information. It would seem likely that cognitive rigidity would impair ‘little-C’ creativity. However, there is so far no empirical research on such creativity for people with an ED.

The purpose of this study is to examine whether creativity, as indicated by a test of ‘little-C’ divergent thinking, is reduced for people with an ED. We used Guilford, Christensen, Merrifield and Wilson’s [8] Alternative Uses Task (AUT), in which participants were asked to list as many possible creative uses for a common household item, such as a newspaper, a brick, or a paper clip. Sternberg, Lubart, Kaufman and Pretz [9] pointed out that many researchers into creativity adopted Guilford’s approach and such tests of divergent thinking became the main instruments for measuring creative thinking. In the AUT there is no single correct answer, and participants must branch out and produce as many unique and creative uses as they can (e.g., origami, fly swatter, hat, picnic blanket, napkin etc., for newspaper). Guilford et al. proposed coding the AUT with regard to four aspects of creative thinking: 1) *fluency*, the ability to produce ideas or problem solutions in a short period of time; 2) *flexibility*, the ability to propose a variety of approaches to a problem; 3) *originality*, the ability to produce new ideas; and 4) *elaboration*, the ability to systematize and organize the details of an idea. Thus, using the AUT enabled us to measure and distinguish different aspects of creativity in people with EDs. Based on the research investigating set shifting and EDs, and given that of the four measures of creativity it is flexibility that seems most connected to poor set shifting, we predicted that people with an ED would show less creative flexibility than other people. Doing so would build on the work by Tchanturia and others by generalizing the finding of inflexibility to a very different task to those used to measure set-shifting. By investigating which specific aspects of creativity were related to having an ED we could address the question of how broad may be the observed cognitive rigidity. By looking across the spectrum of disordered eating thoughts we also asked whether the observed lack of flexibility extended beyond those with a diagnosed ED.

Understanding the relationship between EDs and creativity is important both for helping to understand EDs and because it may have implications for treatment. Tchanturia, Lloyd and Lang [10] have developed Cognitive Remediation Therapy (CRT) for AN as a therapy that tries to encourage more flexible thinking and thus addresses a maintenance factor that may make other AN therapies less effective. They point out that although CRT was developed for use with people with brain injuries it has been used with a range of disorders. Tchanturia, et al. described CRT as a brief individual intervention which encourages patients to discover how they think and to experiment with different ways of thinking and behaving between sessions. They summarize evidence that CRT can be effective in leading AN patients to think more flexibly, and such changes to thinking could be helpful in increasing the effectiveness of other interventions trying to change patient behaviour. However unlike CRT, the set-switching studies that underpin the argument that an ED is associated with less flexibility present participants with defined options rather than ask participants to find new options. In the alternative uses task participants do not demonstrate flexibility by switching between defined categories, they instead do so by switching to new categories they themselves define. Therefore if an ED is associated with lower flexibility in the AUT then this strengthens the theoretical underpinnings of CRT.

## Method

### Participants

We recruited 112 undergraduate women students (mean age of 19.7 years) from a psychology course’s participant pool at a major metropolitan Australian university. By recruiting a non-clinical sample we were able to examine any relationship between creativity and disordered eating across the spectrum of ED symptomology. Amongst these participants, 16 participants had a current or former ED diagnosis. This research was approved by the University of Sydney Human Ethics Office and all participants gave informed consent.

### Materials and procedure

All participant pool members completed pre-screening instruments which included the SCOFF (Morgan, Reid, & Lacey, [11]) for ED. A score of two or more on the SCOFF indicates a possible ED diagnosis. In order to increase the number of participants in our sample with ED symptoms we recruited two equal-sized groups based on their SCOFF score. One group had SCOFF scores less than two and the other group had SCOFF scores equal to or greater than two.

We gave participants the 36 item EDE-Q (Fairburn & Beglin [12]) to assess disordered eating. This scale was derived from the Eating Disorders Examination (EDE, Fairburn & Cooper [13]) and good evidence of its reliability and validity was reported in Berg, Peterson, Frazier and Crow’s [14] systematic review. To assess participants’ creativity the AUT was administered. In our version of the AUT participants were given 3 min to list as many alternative uses for a paper clip as they could. Subsequently participants were asked if they had a current or previously diagnosed ED, but were not required to reveal the diagnosis.

## Results

The mean EDE-Q score for our participants was 2.48 (*SD* = 1.47, range 0.22 to 5.74) and their mean BMI was 21.54 (*SD* = 3.00, range 15.76 to 31.02). Although four subscales have been proposed for the EDE-Q, only global EDE-Q scores were used. Berg et al. [14] reported that studies have repeatedly failed to replicate the four-factor structure that defines the EDE-Q subscales.

The AUT was scored by two coders who were blind to the EDE-Q scores and diagnosis status of participants. They had high inter-rater reliabilities for the four measures with correlations between *r* = 0.90 and *r* = 0.97. Coders first identified which specified uses were *standard uses* (e.g., holding papers together; *M* = 1.34, *SD* = 1.59) and which were *creative uses* (e.g., electrical wire; *M* = 6.10, *SD* = 3.34). Number of creative uses is the typical measure of *fluency*. To measure *originality* participants were given two points per use generated by less than 1% of participants, and one point per use generated by fewer than 5%. Someone with higher fluency is likely to score higher on originality simply because they generated more items, so originality was corrected by dividing it by *fluency*. *Flexibility* was measured by determining the number of categories each participant’s uses fell into. For example, if a participant suggested using paper clips for a necklace and for a bracelet, then these two items fell into the same category (“jewellery”). *Elaboration* is a representation of the level of detail and development of the idea behind the use. It is created by assigning each answer points corresponding to the amount of detail present in the answer.

Table 1 presents the correlations between these measures of creativity and EDE-Q scores across all participants. It shows that across the full range of EDE-Q scores there was no evidence of a relationship between degrees of disordered eating and any of the measures of creativity.

**Table 1:** Correlations between EDE-Q scores and each measure derived from the alternative uses task (AUT)

	Fluency	Originality (corrected)	Flexibility	Elaboration
EDE-Q	.039	.006	−.110	.138
Fluency		.271*	.746*	.589*
Originality			.309*	.458*
Flexibility				.389*

Correlations with an asterisk are statistically significant at the 0.05 level

### Analysis of self diagnosed participants

We then examined participants with a current or former diagnosis of an ED. Six had a current diagnosis (*Mean* EDE-Q = 4.61, *SD* = 0.863) and ten indicated a previous diagnosis (*Mean* EDE-Q = 3.48, *SD* = 1.33). Table 2 presents the mean scores for each AUT creativity measure for the diagnosed and never diagnosed. This shows a tendency for the diagnosed participants to perform more poorly on measures of creativity but to determine which differences were statistically significant we ran a logistic regression analysis on the category diagnosed versus undiagnosed with the four AUT measures entered as predictors. Logistic regression is the appropriate way to analyse the effects of multiple linear predictors on a categorical outcome (Field [15]). A statistically significant model was found, *X* ^*2*^(4) = 13.66, *p* = .008, *R* ^*2*^ = .120 (Cox & Snell) .216 (Nagelkerke). Over dispersion was not present (*ϕ* = 0.916) and there was no evidence of violation of linearity with the logit. Table 3 presents the results of the logistic regression analysis and shows that only flexibility was a statistically significant predictor of whether or not a participant had received a diagnosis. Even though all the measures derived from the AUT are correlated, the flexibility measure accounts for significant variance in the diagnosed variable over and above the other AUT measures. If the other three measures are first entered into the regression equation this yields a nonsignificant overall model, *X* ^*2*^(3) = 5.459, *p* = .141. However adding flexibility into the equation significantly increased its fit to the data, *ΔX* ^*2*^(1) = 8.204, *p* = 0.004.

**Table 2:** Means for participants reporting a diagnosis and those reporting having never been diagnosed for each measures from the alternative uses task

	Creative uses	Originality (corrected)	Flexibility	Elaboration
Diagnosed (*n* = 16)	4.63 (SD = 2.63)	.0922 (SD = .1686)	1.81 (SD = 0.911)	6.50 (SD = 4.94)
Never diagnosed (*n* = 95)	6.38 (SD = 3.39)	.1158 (SD = .2063)	2.92 (SD = 1.29)	7.29 (SD = 4.36)

**Table 3:** Results of logistic regression on diagnosis category (whether or not participant reported having received a diagnosis)

	B	S.E.	Wald	*df*	*p* level	Odds ratio
Constant	.496	.811	.374	1	.541	1.643
Fluency	.083	.182	.207	1	.649	1.086
Elaboration	.042	.092	.208	1	.649	1.043
Originality (corrected)	.197	2.108	.009	1	.926	1.218
Flexibility	−1.286	.504	6.512	1	.011	.276

That this difference in flexibility is associated with diagnosis is illustrated by the distribution of the flexibility measure. It was observed that 63.2% of participants reporting having never been diagnosed used three or more categories in their creative answers, but only 12.5% of those who reported a diagnosis did so.

### Analysis of participants with potential diagnoses

Although only 16 participants reported a current or former ED diagnosis, a number of other participants had high enough EDE-Q scores to suggest a potential ED diagnosis. The EDE-Q is not used for diagnosis by itself, but these high scores suggest it is possible that some participants had an undiagnosed ED or chose not to report a diagnosis to us. So the number of participants with an ED may be higher than we have reported, therefore we tried to use EDE-Q scores to identify participants with a potential diagnosis.

Whereas there is not a published validated procedure for using the EDE-Q alone to diagnose someone with an ED, there have been studies that have examined what level of EDE-Q global score best discriminates between people with or without an independently confirmed ED diagnosis. Mond, Hay, Rodgers, Owen, and Beumont [16] found the criteria that best discriminated diagnosed from undiagnosed participants was having an EDE-Q score above 2.3 plus objective binge eating or exercising to control weight, whereas Mond, Myers, Crosby, Hay, Rodgers, Morgan, Lacey & Mitchell [17] found an EDE-Q greater than 2.8 to be the best discriminator. These two studies were conducted in Australia but Aardoom, Dingemans, Slof Op’t Landt and Van Furth’s [18] large Dutch study seems consistent with 2.8 being a good discriminator. In their study less than 5% of undiagnosed participants had an EDE-Q score of greater than 2.8, and more than 80% of those with a diagnosis had an EDE-Q score greater than 2.8. So we split our data into participants with EDE-Q scores above or below 2.8. By this criterion 47 participants were identified as having a potential diagnosis.

The same logistic regression analysis as used previously was run except for the dependent variable now being potential diagnosis (EDE-Q score above 2.8 versus below 2.8). The model was not statistically significant, *X* ^*2*^(4) = 7.66, *p* = .105, *R* ^*2*^ = .068 (Cox & Snell) .092 (Nagelkerke). However, the results of the analysis (presented in Table 4) show that in the regression equation only the beta for flexibility approached statistical significance.

**Table 4:** Results of logistic regression on having or not having a potential diagnosis based on EDE-Q score

	B	S.E.	Wald	*df*	*p* level	Odds ratio
Constant	−0.372	0.557	0.446	1	.504	0.689
Fluency	0.115	0.104	1.220	1	.269	1.122
Elaboration	0.093	0.064	0.064	1	.145	1.098
Originality (corrected)	−0.97	2.108	0.006	1	.940	0.907
Flexibility	−0. 478	0.257	3.446	1	.063	0.620

## Discussion

Overall we did not find a relationship between self-reported or potential ED diagnosis and creativity overall, except that creative flexibility predicted self-reported ED diagnoses. When we expanded from self-reported diagnoses to potential diagnoses based on EDE-Q scores there was no statistically significant relationship between creativity, just as there were no statistically significant correlations between creativity measures and EDE-Q scores across the whole range of scores. If participants reporting a diagnosis are more likely to have an ED than those simply meeting an EDE-Q criterion, then this suggests that only those with the strongest eating disorders symptoms showed reduced flexibility. This results also leaves open the possibility that receiving a diagnosis, and potentially treatment, of an ED reduced participants’ flexibility. However the logistic regression analysis on potential diagnoses although not statistically significant, found that the only aspect of creativity that came close to statistical significance was flexibility. Further research may be necessary to clarify what level or type of ED symptoms may be associated with lowered flexibility, or whether the effect is only present for people who have received a diagnosis. We also must be cautious in interpreting the failure to reject the null hypotheses of no effect of other aspects of creativity.

However the suggested relationship between lower flexibility and reported diagnosis is in line with previous research showing poor set shifting in those suffering from an ED. To the extent that ED and autism are associated (as suggested by Hambrook, Tchanturia, Schmidt, & Treasure [19]), our results are somewhat convergent with Best, Arora, Porter and Doherty [20] who found that degree of subthreshold autistic traits was associated with less flexibility but not less originality.

We found no relationship between any of the measures of creativity and EDE-Q scores across the full range of disordered eating in our sample of female university students. This is consistent with the meta-analysis conducted by Westwood, Stahl, Mandy, and Tchanturia [5] indicating that cognitive rigidity may be a consequence of developing an ED instead of being a precursor trait prior to the development of an ED. If cognitive rigidity was a precursor of an ED we would expect the degree of ED symptoms (as indicated by EDE-Q scores) that do not reach the level of diagnosis to predict flexibility.

Our finding that creative flexibility, but not originality, was associated with reporting an ED diagnosis helps to build on the previous findings of cognitive rigidity in ED patients and to clarify its scope. By finding less use of different approaches in the AUT we showed that this inflexibility extends beyond having to switch to a given task and appears to extend to situations in which people with an ED need to generate new approaches. However our findings also seem to limit the scope of the cognitive rigidity of diagnosed ED participants. Such participants appeared to be able to think differently enough to generate original thoughts and elaborate on them to a similar extent as participants not reporting an ED diagnosis, but they were less likely to have tried to do the task they were given in many different ways by using different categories. So in this way the cognitive rigidity of people with EDs may be more of a strategic preference than a consequence of the inability to think flexibly. A possible explanation for this could be because people with ED generally have an elevated fear of failure and greater perfectionism (Bulik, Tozzi, Anderson, Mazzeo, Aggen, & Sullivan [21]). Sticking to an approach that works may be a way to avoid the possibility of failure rather than a sign of less creativity. Consistent with this may the finding that in at least some of the studies of set-switching deficits in people with ED the deficit is not in the measure of mistakes but instead in the measure of speed. For example, Tchanturia, Anderluh, Morris, Rabe-Hesketh, Collier, Sanchez, and Treasure [22] and Tchanturia, Morris, Anderluh, Collier, Nikolaou, and Treasure [23] gave people with AN or bulimia nervosa or who were healthy controls a range of tasks for assessing set-shifting tasks with measures of performance based on both speed of responding and based on number of errors. Their results consistently showed effects on measures of speed but not errors. Slowing down when having to switch tasks may be a way to avoid errors. As such, not deviating from a currently productive strategy may be the result of this fear of failure as opposed to an inability to think differently.

Practically, our findings were supportive of the theoretical underpinnings of CRT (Tchanturia et al. [10]) being applied to people with EDs. The results were supportive because they found evidence of lower flexibility in people with EDs, and they also argue that therapy has the opportunity to be effective. If people with an ED lacked the ability to think differently, then it would probably be harder for therapy to be effective at increasing their flexibility. If inflexibility is more a strategy than the result of not being able to come up with different ways to deal with a task then it should be easier to change through therapy, and to some extent CRT could be seen as targeting such strategies. Added evidence of the changeability of flexibility was that it did not seem to be associated with degree of disordered eating across the whole spectrum, instead only distinguishing participants with a reported diagnosis from those without. If such inflexibility is strategic then this could guide what aspects of CRT may be best utilised alongside standard treatment protocols to reduce cognitive rigidity as a maintenance factor in those with EDs.

### Limitations

The strength of our conclusions is tempered by three important limitations of the current study. First, we recruited our participants from a highly selective university, which may have lessened any differences in creativity. Perhaps students at such a university would need to have overcome any cognitive deficits simply in order to be able to perform well enough academically to gain admission. Therefore our results may be limited to this population. Second, the specific diagnosis was unknown for those who self-reported a current or previous ED diagnosis or for those who had a potential diagnosis based on EDE-Q score. Therefore we cannot distinguish between types of EDs, so we do not know whether our findings apply across the spectrum of EDs or are limited to only some types. A third limitation was that although the AUT is a common measure of creativity, it is not the only one and it embodies a certain approach to understanding creativity. Sternberg et al. [9] discussed other approaches. So our conclusion that creativity (other than flexibility) was not associated with having an ED does not preclude the possibility that other measures of creativity may find different results.

## Conclusion

Our results were consistent with previous findings of an association between cognitive inflexibility and EDs, but they suggest that this inflexibility may be limited in scope. We found no evidence that cognitive inflexibility generalized to creativity more broadly, and we only found evidence of differences for participants reporting a current or former ED diagnosis. However they may lend support to Cognitive Remediation Therapy because it is applied within these limitations (i.e., it focuses on increasing the flexibility of thinking of people with an ED diagnosis). The limitations of our study and its many null findings suggest though that further study is needed to clarify these issues.
